# The evaluation of relationship between blood pressure and dialysate Na concentration in chronic hemodialysis patients

**DOI:** 10.15171/jrip.2016.25

**Published:** 2016-05-09

**Authors:** Narges Sadat Zahed, Omid Gharooi, Latif Gachkar, Hajar Nikbakht

**Affiliations:** Department of Nephrology, Loghman Hakim Clinical Research Development Center, Shahid Beheshti University of Medical sciences (SBUM), Tehran, Iran

**Keywords:** Blood pressure, Dialysis, Dialysate sodium concentration, Hemodialysis, Intradialysis weight gain

## Abstract

**Introduction:** Hypertension is one of the traditional risk factors of cardiovascular disease (CVD). Extra cellular volume expansion and Na retention remain the main cause of hypertension.

**Objectives:** The aim of this study was to investigate the relation between concentration of Na dialysate and blood pressure (BP) in chronic hemodialysis (HD) patient.

**Patients and Methods:** This cross-sectional study was performed on 266 adult patients undergoing HD for at least three months. Pre-HD systolic BP (SBP) and post-HD SBP during 4 weeks were measured in relation to Na dialysate concentration. The other main factors affecting the post-dialysis BP, such as body mass index (BMI), pump speed, dialysis solution temperature, duration of dialysis and intradialysis weight gain (IDWG) were also considered. Mean of ΔSBP (post-HD SBP – pre-HD SBP) in each patient in 12 session of HD was measured and statistically analyzed in relation to dialysate Na with SPSS 21. Backward multivariable linear regression analysis and Pearson’s correlation coefficients were used to evaluate the correlation between sodium gradient and ΔSBP.

**Results:** SBP was significantly changed before and after dialysis in relation to dialysate Na (*P*<0.001). The Pearson’s correlation between ΔSBP with dialysate sodium and blood flow rate (pump speed) were statistically significant(*P*<0.05).

**Conclusion:** We found that changes in SBP before and after dialysis is significantly associated with dialysate sodium concentration.

**Implication for health policy/practice/research/medical education:**

About 50% to 90% of dialysis population have hypertension. A relation between chronic volume expansion and mortality is well established. Dialysate sodium concentration is proposed as one of several factors which can control the blood pressure (BP) in dialysis patients. The aim of this study was to investigate the relation between concentration of Na dialysate and BP in chronic hemodialysis (HD) patients. This cross-sectional study was performed on 266 adult patients undergoing HD for at least three months. We found that changes in systolic BP before and after dialysis is significantly associated with dialysate sodium concentration.

## Introduction

Cardiovascular complications and more specifically hypertension are the most common causes of death in chronic dialysis patients (1). About 50% to 90% of dialysis population have hypertension (2). According to a cohort study that was performed by Agarwal et al on 2535 patients, 86% of them had hypertension although after antihypertensive medication in 76% of patients only 30% of cases had an appropriate responds to treatment (3).

Extracellular volume expansion and Na retention remain the main causes of hypertension in end-stage kidney failure (ESKF). A relation between chronic volume expansion and mortality is well established dialysate sodium concentration is proposed as one of several factors which can control the blood pressure (BP) in dialysis patients (4-6).

The first dialysate sodium concentration was 126.5 meq/L (Kolff 1974). In order to prevent further complications such as sudden decrease in serum osmolality, muscle cramps and hypotension during dialysis, the dialysate sodium concentration increase gradually to 135-145 meq/L. Although increasing dialysate sodium concentration, can lead to thirst, increased BP and weight gain during the dialysis. Davenport et al conducted a study on 469 dialysis patients, which concluded that lower sodium dialysate can gain BP control (4). Though the mentioned work is in contrast with investigation among 2187 patients in 2008, which did not show the relationship between BP and dialysate sodium concentration (5). Krautzig et al could reduce the mean arterial pressure from 108 to 98 by decreasing the dialysate sodium concentration from 140 to 135 in a period of 15 to 20 weeks (6). In contrast, Kooman et al were unable to decrease BP by reducing dialysate sodium from 140 to 136 (7). Sayarlioglu et al selected 135 meq/L dialysate Na concentration for patients with serum sodium lower than 137 meq/L and 137 meq/L dialysate sodium concentration for patients with serum sodium higher than 137 meq/L. In the same study a significant reduction in pre-dialysis systolic BP (SBP) (151.7±17.7 versus 179±24.8) and post-dialysis SBP (132.3±16.4 versus 141.4±28.8) were observed after 8 weeks (8). Finally, the large scale study on 23962 dialysis patients carried out in 2014 shows no significant difference in pre-dialysate SBP between patients with typical dialysate sodium concentration of 135 meq/L to 145 meq/L and patients with individualized dialysate sodium concentration of 125 meq/L to 155 meq/L (9). The different results obtained by different groups convinced the authors to conduct the current work among 266 patients to reexamine the effect of dialysate sodium concentration on SBP in hemodialysis (HD) patients.

## Objectives

The aim of this study was to investigate the relation between concentration of Na dialysate and BP in chronic HD patients.

## Patients and Methods

In this observational study, patients were enrolled from three different dialysis centers from educational hospital in Tehran including Loughman Hakim hospital, Ashrafi Isfehani hospital and the West center.

All selected patients were undergoing HD three times per week (4 hours each session) for more than two months until they reach to their dry weight. Then, 266 dialysis patients were enrolled in the study.

The project is performed by nephrologists and dialysis staff. This investigation is not considered as human subject research, because of patients undergoing ordinary treatment. The dialysis is performed for all participants with volumetric dialysis machines (JMS, SD 50).

The standard dialysis prescription included session length of dialysis (about 4 hours) with individual blood flow rate based on patients’ condition (from 210 cc/min to 350 cc/ min) and dialysate flow rate of 500 mL/min. The dialysis solution contains the following concentration: potassium, 1-3 meq/L; bicarbonate, 35-40 meq/L; magnesium, 1.0 meq/L; and calcium, 2.5 meq/L. Glucose concentration was 200 mg/dl.

BP is monitored before and after the dialysis with calibrated mercury sphygmomanometer in each session by dialysis nurse during 4 weeks.

All data including patient age, duration of ESKD, etiology of ESKD, pre- and post-dialysis weight, amount of ultrafiltration (UF), pre- and post-dialysis BP, blood flow rate (pump speed), dialysis temperature, body mass index (BMI) and medication such as antihypertensive drugs were collected in data sheets. Dietary Na intake was not measured. In patients with high BP (define as BP > 140/90 mm Hg) sodium dialysate decreased to 133 meq/L, and patients with low BP (define as BP <100/80 mm Hg), Na dialysate increased to 147 meq/L.

Moreover, the patient’s medications such as anti-hypertensive drugs are not modified.

We predict the post-dialysis BP with the following formula:

SBP after dialysis – SBP before dialysis = -41.5 + 0.11 (sodium of dialysate) – 0.3 (speed of pump).

### Ethical issues

The research followed the tenets of the Declaration of Helsinki; informed consent was obtained; and the research was approved by the ethical committee of Shahid Beheshti University of Medical Sciences.

### Statistical analysis

**Figure 1:**
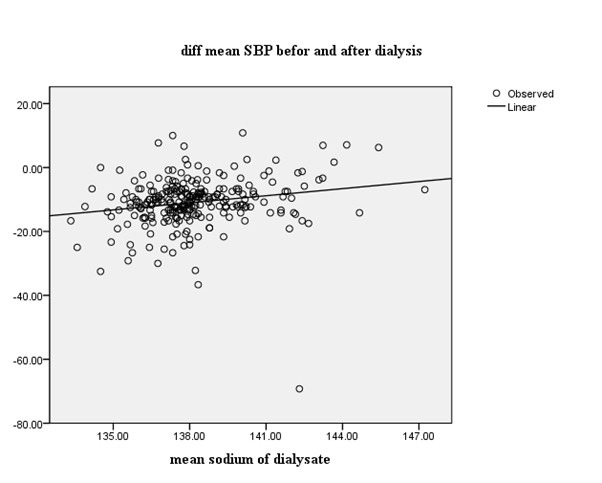
Systolic blood pressure (SBP) and mean sodium concentration of dialysate.

**Figure 2:**
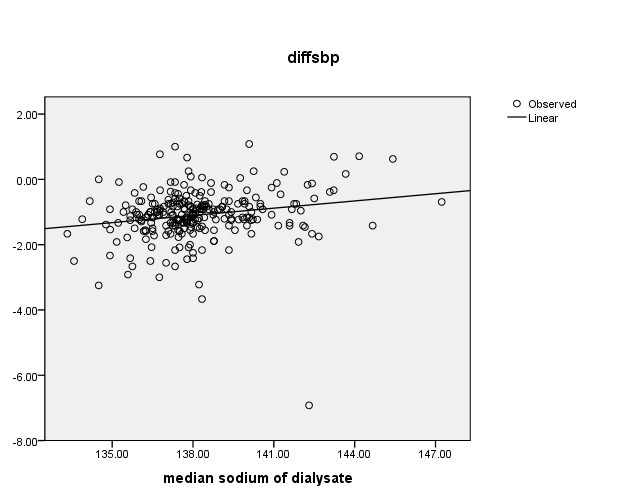
Systolic blood pressure (SBP) and median sodium concentration of dialysate

Data variables are summarized as mean ± standard deviation (SD) or median with interquartile range. Stepwise multivariable linear regression and Pearson’s correlation coefficient was used to evaluate the correlation between post- and pre-dialysis BP with independent variables such as dialysate sodium concentration, intradialysis weight gain (IDWG ), duration of dialysis and dialysis fluid temperature. Post-dialysis BP predicted by backward multivariable linear regression analysis (Figures 1 and 2). A *P* value <0.05 was recognized statistically significant.

## Results

A total of 266 HD patients were studied (153 were men). Mean age was 57.97±13.33 years and 71 (26.6%) had diabetes, 98 (36.8%) had hypertension. Mean pre- and post-HD-BP were 127.14±17.55 mm Hg, and 116.23±14.85 mm Hg, respectively. Mean Na dialysate was 138.16±2.06 meq/L, IDWG 2.18±0.91 kg, dialysis fluid temperature 36.28±0.29°C, pump speed 276.17±21.47 cc/min, duration of sessions 3.91±0.22 hours (TTables 1 and 2). In the second step, delta of SBP (ΔSBP), post-HD SBP - pre-HD SBP was calculated. Analysis performed in six steps. In stepwise multiple regression model, ΔSBP was the dependent variable, and the other variable were independent. At second step dialysis temperature, in the third step BMI, months’ of dialysis in fourth step, duration of dialysis in fifth and in sixth step and IDWG were excluded (Table 3).

**Table 1:** Etiology of ESKF in dialysis patientsa

**Etiology ESKD**	**Prevalence**
Diabetes	71 (26.6)
BP	98 (36.8)
Polycystic kidney	12 (4.5)
Kidney stone	10 (3.8)
Undermined	10 (3.8)
Missed data	65 (24.4)

Abbreviations: BP: Blood pressure; ESKF, end-stage kindney failure. ^a^N = 266. Values are expressed as number (percentage).

**Table 2:** Mean ± SD of variables

**Variable**	**Min**	**Max**	**Value**
Age (years)	25	83	57.97 ± 13.33
BMI( kg/m^2^)	15	43	24.96 ± 3.65
Dialysis duration (month)	2	312	49.01 ± 46.91
Pre dialysis systolic pressure (mm Hg)	69.2	203.8	127.14 ± 17.55
Post dialysis systolic (mm Hg)	65.4	167.5	116.23 ± 14.85
Dialysis pumps speed (m/min)	210	325.56	276.17 ± 21.47
Dialysis solution temperature (C)	35	36.91	36.28 ± 0.29
Dialysis session duration (h)	2.43	4	3.91 ± 0.22
Interadialytic weight gain (kg)	0.06	4.65	2.18 ± 0.91
Dialysate sodium concentration (mEq/L)	133.33	147.27	138.16 ± 2.06

**Table 3:** Variables partial correlation

**Model **	**Standardized coefficients beta **	**t **	***P***	**Partial Correlation **	**Collinearity Statistics**
					**Tolerance**
2	Mean temperature of dialysate	-0.007^a^	-0.111	0.912	-0.007	0.813
3	Mean temperature of dialysate	-0.008^b^	-0.127	0.899	-0.008	0.823
	Body mass index	0.009^b^	0.148	0.882	0.009	0.890
4	Mean temperature of dialysate	-0.009^c^	-0.138	0.890	-0.009	0.824
	Body mass index	0.005^c^	0.086	0.931	0.005	0.909
	Months of dialysis	0.025^c^	0.412	0.681	0.026	0.956
5	Mean temperature of dialysate	-0.011^d^	-0.175	0.862	-0.011	0.831
	Body mass index	0.004^d^	0.071	0.944	0.004	0.911
	Months of dialysis	0.023^d^	0.396	0.693	0.025	0.958
	Mean time of dialysis	-0.024^d^	-0.399	0.691	-0.025	0.951
6	Mean temperature of dialysate	0.010^e^	0.167	0.868	0.010	0.913
	Body mass index	-0.015^e^	-0.252	0.801	-0.016	0.994
	Months of dialysis	0.021^e^	0.359	0.720	0.022	0.959
	Mean time of dialysis	-0.029^e^	-0.487	0.627	-0.030	0.957
	Mean weight gain between dialysis	-0.070^e^	-1.110	0.268	-0.069	0.837

^a^Predictors in the Model: (Constant), mean time of dialysis, months of dialysis, mean speed of pomp, body mass index, mean sodium of dialysate, mean weight gain between dialysis. ^b^Predictors in the Model: (Constant), mean time of dialysis, months of dialysis, mean speed of pomp, mean sodium of dialysate, mean weight gain between dialysis. ^c^Predictors in the Model: (Constant), mean time of dialysis, mean speed of pomp, mean sodium of dialysate, mean weight gain between dialysis. ^d^Predictors in the Model: (Constant), mean speed of pomp, mean sodium of dialysate, mean weight gain between dialysis. ^e^Predictors in the Model: (Constant), mean speed of pomp, mean sodium of dialysate. ^f^Dependent Variable: diff mean SBP before and after dialysis.

**Table 4:** Pearson’s correlation

	** **	**Difference mean SBP before and after dialysis**	**Mean sodium of dialysate**	**Mean weight gain between dialysis**	**Mean speed of pomp**
Difference mean SBP before and after dialysis	Pearson’s correlation	1	0.197**	-0.095	-0.321**
	Sig. (2-tailed)		0.001	0.122	0.000
	N	266	266	266	266
Mean sodium of dialysate	Pearson’s correlation	0.197**	1	0.236**	-0.291**
	Sig. (2-tailed)	0.001		0.000	0.000
	N	266	266	266	266
Mean weight gain between dialysis	Pearson’s correlation	-0.095	0.236**	1	0.244**
	Sig. (2-tailed)	0.122	0.000		0.000
	N	266	266	266	266
Mean speed of pomp	Pearson’s correlation	-0.321**	-0.291**	0.244**	1
	Sig. (2-tailed)	0.000	0.000	0.000	
	N	266	266	266	266

**Correlation is significant at the 0.01 level.

With this model variables with low significancy were excluded (IDWG, dialysate temperature, BMI, duration of dialysis). In the final step only dialysate Na concentration and pump speed remained (*P*<0.01). A significant positive correlation between delta SBP and dialysate Na was found. These findings imply that, BP level was significantly different before and after dialysis in relation to dialysate Na (*P*<0.001; Figures 1 and 2).

Additionally the correlation between ΔSBP and dialysate sodium and pump speed was statistically significant (*P*<0.05).

## Discussion

BP depends on heart output and peripheral vascular resistance. Increased dietary sodium intake results in higher plasma sodium concentrations. If sodium intake exceeds the capacity of renal excretion plasma volume and BP will increase (10).

Moreover, it is demonstrated that the limitation in daily salt intake to the amount of 4.4 to 7.4 gram/day can reduce SBP by 3.7 to 4.9 mm Hg and diastolic BP by 0.9 to 2.9 mm Hg (10).

It should be noted that all factors which reduce plasma sodium concentration, could contribute to reduction of BP in hemodialysis patients too.

Extracellular volume expansion and Na retention remain the main causes of hypertension in HD patients. There is an association between extracellular fluid volume (ECFV) expansion and diastolic dysfunction in dialysis patients. Recent attention has been called to none osmotic accumulation of Na in subcutaneous space and other organs, elevated Na stores may impact inflammatory and cardiac fibrotic process via vascular endothelial growth factor. Also Na accumulation in arterial smooth muscle may contribute to increased vascular stiffness (11).

In HD patients most of Na intake is driven by salt ingestion, another source of Na is diffusive gain from dialysis solution when dialysate sodium is greater than the pre-dialysis plasma level. The difference between plasma and dialysate sodium concentration induces sodium gradient across dialysis membrane. Sodium concentration in the dialysate solution should be 6 to 7 meq/L higher than the plasma sodium concentration to obtain isonatremic dialysis. In fact, Gibss-Donnan effect compensates this gradient in vivo. Gibss-Donnan effect is due to a large portion of the negative charge in the plasma protein which is not able to pass through dialysis membrane. This effect induces 4% to 5% reduction in sodium diffusion, itself (12). In a study on 2187 patients, conducted by Davenport et al, for one week, very little change in the BP was observed and, therefore, it failed to prove the relationship between sodium dialysate concentration and BP (5). Whereas in investigations by Elshahawy et al and Movilli et al, during a period of at least six months, statistically and clinically significant relationship was achieved (13,14). Assessing Na kinetics during a dialysis session by sequential plasma and dialysate Na measurement is impractical in clinical practice, therefore, we decided to asses post-dialysis BP just in relation to Na dialysate (dietary Na intake and plasma Na were not measured).

We found that if dialysate Na is decreased about 13.3 meq/L (from 147.33±2.06 to 133.33±2.06) BP will decrease about 10.91 mm Hg. This positive correlation is very important because we know with this strategy not only we can control BP in hypertensive patients, but also we can predict post-dialysis BP according to Na dialysate and pump speed.

We also found out that IDWG has positive correlation with Na dialysate (Table 4). IDWG is not only important in control of BP in hypertensive patients but also it is important in decreasing of BP in hypotensive patients (15).

Another important factor is pump speed, if pump speed decreases from 210 cc/min to 325.56, the BP will decrease significantly (*P*<0.001).

We explained that differences in our results with other studies may be due to the duration of follow up. In the study of Davenport et al, was done only for one week, duration of study was too short, whereas changes in Na concentration should be made gradually over the time in order to changes in ECFV, and storing of Na in body.

## Conclusion

We found post-dialysis BP is significantly correlated with changes in dialysate sodium concentration. This Relationship is independent of other clinical factors.

## Limitations of the study

The limitations of our stuy was small sample size and short duration of investigation. Moreover, our study was observational, while prospective studies in order to predict BP in relation to Na accumulation and its consequences is needed.

## Acknowledgments

We woulde like to acknowledge Loghman-Hakim Clinical Research Development Center because of its kind supports.

## Authors’ contribution

NSZ; has designed the idea of study, wrote the discussion part, and observed accuracy and validity of study protocol. OG; collected data and fallow the studies objects. LG; performed statistical analysis. HN wrote the article paper and edited the manuscript.

## Conflicts of interest

The authors declare no conflict of interest.

## Ethical considerations

Ethical issues (including plagiarism, data fabrication, double publication) have been completely observed by authors.

## Funding/Support

This article is extracted from residential thesis of Omid Gharooi. This study was supported by a grant from Shahid Beheshti University of Medical Sciences (Grant # 55, 2010).
